# Efficacy and acceptability of parent-only group cognitive behavioral intervention for treatment of anxiety disorder in children and adolescents: a meta-analysis of randomized controlled trials

**DOI:** 10.1186/s12888-020-03021-0

**Published:** 2021-01-11

**Authors:** Bangmin Yin, Teng Teng, Lyu Tong, Xuemei Li, Li Fan, Xinyu Zhou, Peng Xie

**Affiliations:** 1 Department of Neurology, People’s Hospital of Deyang City, Deyang, China; 2 Department of Neurology, The First Affiliated Hospital of Chongqing Medical University, Chongqing, China; 3 NHC Key Laboratory of Diagnosis and Treatment on Brain Functional Diseases, The First Affiliated Hospital, Chongqing Medical University, No. 1 Youyi Road, Chongqing, 400016 China; 4 Department of Immunology, College of Basic Medical Sciences, China Medical University, Shenyang, China; 5 Department of Psychiatry, The First Affiliated Hospital of Chongqing Medical University, No. 1 Youyi Road, Chongqing, 400016 China

**Keywords:** Anxiety, Parent-only CBT, Meta-analysis, Child, Adolescent

## Abstract

**Background:**

Anxiety disorder is the most prevalent mental disorder among children and adolescents, causing significant psychosocial problems and physical health conditions. Cognitive behavioral therapy (CBT) is an effective treatment for anxiety disorder in children and adolescents. And parent-only CBT is an alternative treatment for childhood anxiety disorder, which includes psychologists and parents rather than children in the treatment. As a new type of CBT, parent-only CBT has some advantages. However, it remains unclear whether parent-only CBT interventions are effective for treating children and adolescents with anxiety disorder.

**Methods:**

In this study, we evaluated the efficacy (the mean change scores of the anxiety rating scale from baseline to post-treatment, standardized mean difference SMD) and acceptability (the proportion of patients in the treatment group who withdrew from treatment early for any reason, risk ratios RRs) of parent-only cognitive behavioral therapy (CBT) for children and adolescents with anxiety disorder. We searched electronic databases, including PubMed, Cochrane Library, Embase, Web of Science, ProQuest, and PsycINFO from inception to June 2019. We included randomized controlled trials (RCTs) comparing parent-only CBT either with waitlist (WL), or CBT with parents in children and adolescents with anxiety disorder.

**Results:**

Finally, six RCTs with 407 participants were included in the meta-analyses. In terms of efficacy, pooled analyses indicated that parent-only CBT was significantly more effective than WL for reducing anxiety symptoms with SMD of − 0.72 (95% CI − 1.41 to − 0.03, *p* = 0.04), and more remission rate with RR of 4.33 (37.96% vs. 6.85, 95% CI 1.82 to 10.27, *p* = 0.0009) at post-treatment. And our analyses showed no evidence that parent-only CBT had significantly greater efficacy than CBT with parents with SMD of 0.21 (95% CI − 0.09 to 0.50, *p* = 0.17). Acceptability in the parent-only CBT group was not significantly different to the WL group with RR of 0.92 (95% CI 0.52 to 1.62, *p* = 0.77), and was significantly worse than in the CBT with parents group with RR of 1.93 (95% CI 1.05 to 3.57, *p* = 0.03).

**Conclusions:**

Current evidence indicates that parent-only CBT can be an alternative and acceptable intervention for treating children and adolescents with anxiety disorder.

**Supplementary Information:**

The online version contains supplementary material available at 10.1186/s12888-020-03021-0.

## Background

Anxiety disorder is the most prevalent mental disorder among children and adolescents. Internationally, 6.5% of all children and adolescents meet the diagnostic criteria for anxiety disorder at least once in their life [1]. Anxiety disorder causes significant psychosocial problems, including impaired academic and social competence, and can lead to physical health problems [2, 3].

Cognitive behavioral therapy (CBT) is an effective treatment for childhood anxiety disorder. And parent-only CBT is an alternative treatment for childhood anxiety disorder, which includes psychologists and parents rather than children in the treatment. By previous study, we reported meta-analysis about child-CBT for anxiety disoder and noticed parent-only CBT for children anxiety is an interesting treatment which have unique advantages, especially for young children. So on the basis, we did this research focused on parent-only CBT [4–10]. As one type of CBT used with children and adolescents, CBT with parents has been demonstrated to be effective for treating children and adolescents with anxiety [5, 11, 12]. However, child-focused CBT has several disadvantages. First, language and cognitive competence are major obstacles, especially for young children [13]. Stigma associated with receiving mental health intervention is another significant obstacle for children [14, 15], as children are more likely to be stigmatized by others for help-seeking behaviors, compared with parents [14]. Furthermore the importance of the family environment and parenting style factors have been identified in previous studies focused on the etiology of childhood anxiety disorders [15].

To respond to these challenges, research into parent-focused interventions for childhood anxiety disorder is increasing. This type of intervention has the potential to avoid the problems mentioned above. However, it remains unclear whether parent-only CBT interventions are effective for treating children and adolescents with anxiety disorder, and previous studies have produced conflicting findings [13, 16–18]. Therefore, we designed a conventional meta-analysis to determine the effectiveness of parent-only CBT to treat anxiety disorder in children and adolescents, compared with WL or CBT with parents as comparison group.

## Methods

### Data sources and searches

PRISMA guidelines [19] were used for conducting this meta-analysis. We conducted a systematic search of six electronic databases: PubMed, Cochrane Library, Embase, Web of Science, ProQuest, and PsycINFO from inception to June 2019. No restrictions were used regarding language. The keywords included: anxiety OR anxious OR phobic OR fear OR fears OR phobia OR phobias, and adolesc* OR child* OR boy* OR girl* OR juvenil* OR minors OR paediatri* OR pediatri* OR pubescen* OR school* OR student* OR teen* OR young, and behavio* OR cogniti* OR CBT OR famil* OR “contingency management”, and parent* OR mother OR father, and random* OR allocate* OR assign* OR “cross over*” OR crossover* OR controlled. More details of literature search reports can be found in Additional file 1. Furthermore, to identify additional eligible randomized controlled trials (RCTs), the reference lists of relevant studies were scanned.

### Studies selection and comparisons design

To determine appropriate studies for our meta-analysis exploring the efficacy and acceptability for treatment of children and adolescents with anxiety disorder, we selected studies according to the following criteria: (1) we included any RCTs investigating the application of parent-only CBT for the treatment of anxiety disorder in children and adolescents, and comparing parenr-only CBT with WL or CBT with parents; (2) we included studies with children and adolescents who were under the age of 18; (3) All participants met a primary diagnosis of a current anxiety disorder, conforming to standardized diagnostic criteria (DSM-IV and K-SADS-PL) [20–22]. to enhance the reliability of our conclusions, we designed two comparisons that complemented each other. One group compared parent-only CBT with WL as a control group, while another group compared parent-only CBT with CBT with parents. In order to focus on anxiety disorder, we excluded studies in which more than 20% of participants had a primary diagnosis of other mental disorders. We adapt relatively strict criterion about including children with anxiety disorder, so studies in which patients were only described with anxiety symptoms rather than confirmed diagnoses were excluded. Because anxiety disorder is frequently comorbid other mental disorders, we did not exclude studies in which participants had a secondary diagnosis of comorbid psychiatric diseases such as major depression and attention deficit hyperactivity disorder. For avoiding influence of drug, trials were excluded if more than 20% of patients took psychotropic drugs. Previous literature introduced Primary Care Behavioral Health (PCBH) model designed by the Veteran’s Administration and the Bureau of Primary Care [23]. The PCBH model recomends that the patients who came to the first consult with the psychologist needs 4–6 sessions CBT. And in accord with our previous study [24], we excluded trials if they met the following criteria: the duration of treatment was less than 6 weeks, or the number of sessions was less than 6. Finally, studies were excluded if we detected repeated publication.

The inclusion process was conducted by two independent reviewers (BY and TT). First, the reviewers scanned the abstract and title of potential papers, and identified studies to be read in full text. The final selection of studies was conducted by both reviewers. If there was any disagreement between the two reviewers, another reviewer was consulted to resolve the discrepancy (XZ).

### Outcome measures

The primary outcome of efficacy was the mean change scores of the anxiety rating scale from baseline to post-treatment. When there was more than one available anxiety rating scale in one study, we used the scores from the anxiety rating scale according to a predefined hierarchy [24], based on psychometric properties and frequency for use with children and adolescents. We also established a hierarchy of informants of anxiety rating scales, with the child or adolescent self-report first in the hierarchy, then the parent/teacher report and then the clinician report. Finally the hierarchy of anxiety symptom severity measurement scales are as below: (1) Revised Children’s Manifest Anxiety Scale (RCMAS), (2) Spence Child Anxiety Scale, Child and Parent Versions (SCAS), (3) Screen for Child Anxiety-Related Emotional Disorders (SCARED), (4), Clinician severity ratings (CSR) (5) Fear Survey for Children Revised (FSSC-R), (6) Child Behavior Checklist (CBCL), (7) other scale.

The second efficacy outcome was remission rate measured by the proportion of participants who did not meet the standardized diagnostic criteria for anxiety disorder after treatment.

The outcome of acceptability was all-cause discontinuation, defined as the proportion of patients in the treatment group who withdrew from treatment early for any reason.

### Data extraction and methodological quality

Two independent reviewers (BY and TT) used standardized data extraction forms to extract data of the main characteristics of all trials, and the methodological qualities of trials were also assessed. Standardized data extraction forms included data on study characteristics (e.g., publication year, first listed author, country, journal, sponsor, institution), intervention details (e.g., duration of treatment, session of treatment, treatment pattern), patients’ characteristics (the number of patients, diagnostic criteria for anxiety disorders), and outcome measures (e.g., remission rate, pre- and post-treatment outcomes). The risk of bias among all studies was assessed according to the Cochrane Collaboration Risk of bias tool described in the Cochrane Handbook for Systematic Reviews of Intervention [25]. If there was any disagreement between the two reviewers, XZ was consulted to resolve the discrepancy. We did not determine the risk of bias across studies, because the number of included studies was too small.

### Statistical analysis

We compared the relative efficacy and acceptability by performing a meta-analysis using Review Manager 5.3 (The Cochrane Collaboration of The Nordic Cochrane Center in Copenhagen). Because of various anxiety rating scales among included studies, there maybe exist underlying heterogeneity and the difference in the true treatment effect among studies. So the DerSimonian and Laird random-effects models were adopted for all meta-analyses [26].

Pooled estimates of standardized mean difference (SMD) were calculated with 95% confidence intervals (CIs) for continuous outcomes. An SMD of more than 0 indicates that the comparison conditions (WL or CBT with parents) were more effective. Conversely, an SMD value of less than 0 indicates that the parent-only CBT condition was more effective. Regarding the risk ratios (RRs), we calculated 95% confidence intervals for discontinuous outcomes. Values of more or less than 1 indicate that the comparison groups or parent-only CBT group more frequently involved events of concern, respectively. Additionally, heterogeneity of both effects among studies was assessed using the *p*-values of the Q statistic and I^2^ statistic. For the primary outcome of efficacy(parent-only CBT compared with WL), we realized significant heterogeneity(I^2^=81%, *P*=0.001). Then we performed sensitivity analysis by excluding the outlier, the study of Cartwright-Hatton (2011) in forest plot Fig. 2a.

To test significant differences in primary efficacy between the different categories of studies, we also performed subgroup analyses. The studies were divided into different categories based on the following factors: male to female ratio (more than 1 or not more than 1), the risk of bias according to the Cochrane Collaboration Risk of bias tool (high bias risk or unclear risk) and the anxiety rating scale (other-rated scale or self-rated scale), respectively.

As for publication bias, we performed Egger’s teston STATA.

## Results

### Study selection and characteristics

**Fig. 1:**
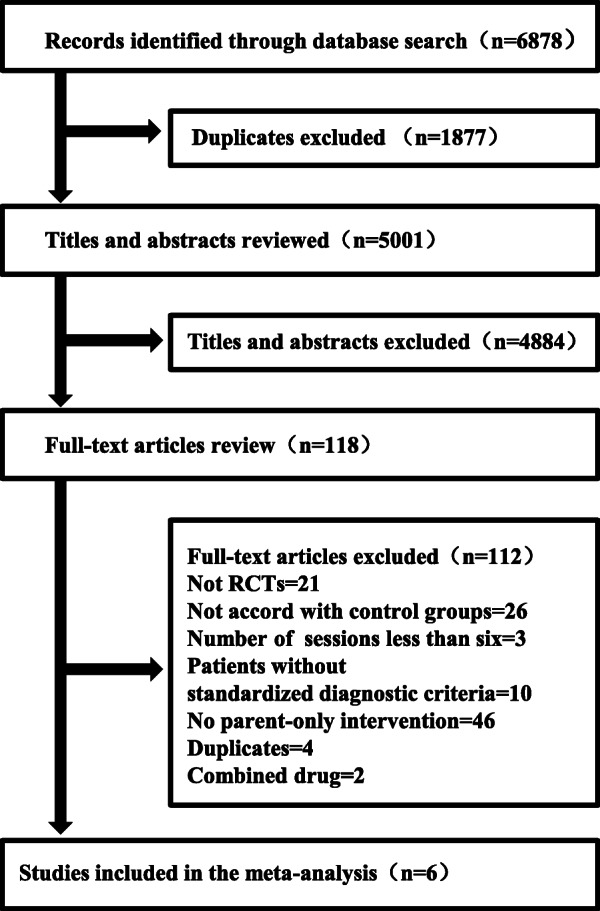
Flowchart of study selection

After searching six electronic databases, we identified 6878 potentially relevant studies. Then, 118 full-text articles were identified for review. Finally, six RCTs with 407 participants were included in our meta-analyses. A flow diagram showing the details of the inclusion and exclusion of studies is presented in Fig. 1. And more details of exclusion can be found in Additional file 2.

Detailed information about the included studies is presented in Table 1 and Additional file 3. There were four studies of parent-only CBT compared with WL, and three studies of parent-only CBT vs. CBT with parents. In total, we included 199 patients in the parent-only CBT group, 114 patients in the WL group and 94 patients in the CBT with parents group. The mean age of all patients recruited was 8.0 years old.

**Table 1:** Characteristics of included studies

Study	Intervention(n)	Diagnosis	Type of Anxiety	Age, Range (Mean)	TreatmentDuration	Treatment Sessions	Anxiety Scales	Primary Efficacy Outcomes	RSG	AC	BPP	BOA	IOD	SR	OB
**Cartwright-Hatton** **2011** **[**18**]**	**parent only CBT(38)** **vs. WL(36)**	**ADIS-IV**	**PD&SAD &SPP&SOP**	**2.7–9(6.6±2)**	**10 w**	**10 Sessions**	**MASC-C**	**0.39 vs.-3.26**	**+**	**+**	**–**	**–**	**+**	**+**	**?**
**Cobham** **2017** **[**13**]**	**parent only CBT(33)** **vs. WL(30)**	**ADIS-IV**	**GAD&SAD&SPP&SOP**	**7–14(9.3±2)**	**6 w**	**6 Sessions**	**SCAS-C**	**−10.5.vs.2.8**	**+**	**?**	**–**	**–**	**+**	**+**	**+**
**Mendlowitz 1999 **[27]	**parent only CBT(21)** **vs. CBT + parent(18)**	**DSM-IV**	**Mixed anxiety disorders**	**7–12(9.8±x)**	**12w**	**12 Sessions**	**RCMAS-37**	**−4 vs. 0**	**?**	**?**	**–**	**–**	**+**	**+**	**+**
**Monga 2015 **[28]	**parent only CBT(32)** **vs. CBT + parent(45)**	**ADIS-IV**	**Mixed anxiety disorders**	**5–7(6.8±0.8)**	**11w**	**11 Sessions**	**SCARED-P**	**−5.73vs.-4.42**	**?**	**?**	**–**	**+**	**+**	**+**	**+**
**Özyurt 2016** **[**29**]**	**CBT parent only(37)** **vs. WL(37)**	**K-SADS-PL**	**GAD&SAD&SPP&SOP**	**8–12(9.7±1.45)**	**8 w**	**8 Sessions**	**SCARED-C-41**	**−12.42 vs.-1.31**	**+**	**?**	**–**	**–**	**?**	**–**	**+**
**Waters 2009** **[**17**]**	**parent only CBT(38)** **vs. CBT + parent(31)vs. WL(11)**	**ADIS-IV**	**GAD&SAD&SPP&SOP**	**4–8(6.8±1.19)**	**10w**	**10 Sessions**	**CSR**	**−4.27 vs.-3.68vs.-0.60**	**?**	**?**	**+**	**+**	**+**	**+**	**+**

*Abbreviations*: *AC* allocation concealment, *ADIS* Anxiety Disorders Interview Schedule for DSM-4, *BOA* blinding of outcome assessment, *BPP* blinding of participants and personnel, *CBT* cognitive-behavioral therapy, *CSR* Clinician severity ratings, *DSM* Diagnostic and Statistical Manual of Mental Disorders, *IOD* incomplete outcome data, *MASC* Multidimensional Anxiety Scale for Children, *OB* other bias, *RCMAS* Revised Children’s Manifest Anxiety Scale, *RSG* random sequence generation, *SADS* Schedule for Affective Disorders and Schizophrenia, *SCARED* Screen for Anxiety and Related Disorders, *SCAS* Spence Children’s Anxiety Scale, *SR* selective reporting, *WL* waitlist

Symbol: -:high risk of bias, +:low risk of bias,?:unclear risk of bias, x:not stated

### Efficacy outcome

**Fig. 2:**
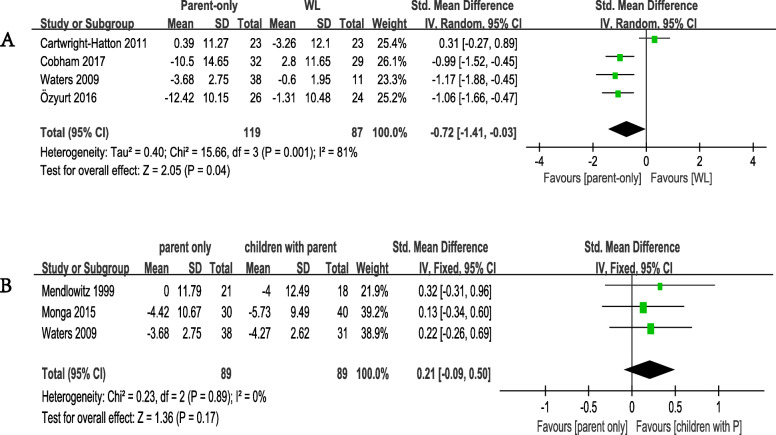
Meta-analysis of primary efficacy outcome. **a** Comparison of parent-only and WL for the primary efficacy outcome: change scores in anxiety rating scales. **b** Comparison of parent-only and children with parent for the primary efficacy outcome

For the primary outcome of efficacy, we first compared the parent-only CBT with the WL control group. The overall pooled SMD indicated a significant advantage when parent-only CBT was compared with WL, with SMD of − 0.72 (95% CI − 1.41 to − 0.03, *p* = 0.04, 4 studies including 206 patients, Egger’s test *P* = 0.787) and high heterogeneity (I^2^ = 81%, *p* = 0.001 Fig. 2a). We then compared parent-only with CBT with parents, with SMD of 0.21 (95% CI − 0.09 to 0.50, *p* = 0.17, 3 studies including 178 patients, Egger’s test *P* = 0.339) and low heterogeneity (I^2^ = 0%, *p* = 0.89 Fig. 2b).

**Fig. 3:**
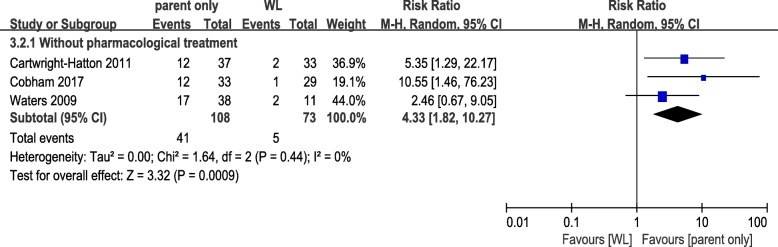
Meta-analysis of secondary efficacy outcome. Comparison of parent-only and WL for the secondary efficacy outcome: the proportion of those who were freed from an anxiety diagnosis at posttreatment

For the secondary outcome of efficacy, only 3 studies including 181 patients reported information about remission rate. All three of these studies belonged to the WL control group. Patients receiving parent-only CBT were more likely to report remission than those receiving WL, with RR of 4.33 (37.96% vs. 6.85, 95% CI 1.82 to 10.27, *p* = 0.0009, Egger’s test *P* = 0.295) and low heterogeneity (I^2^ = 0%, *p* = 0.44 Fig. 3).

### Acceptability outcomes

**Fig. 4:**
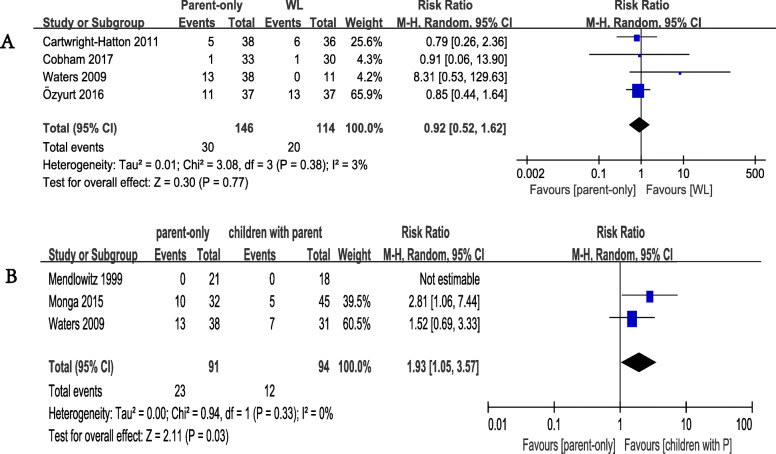
Meta-analysis of acceptability outcomes. **a** Comparison of parent-only and WL for the acceptability outcome: all-cause discontinuation from the trials for any reason. **b** Comparison of parent-only and children with parent for the acceptability outcome: all-cause discontinuation from the trials for any reason

In terms of the acceptability outcome, we found no significant difference between the parent-only intervention group and the WL group. The all-cause discontinuation rates were 30/146 (20.55%) for the parent-only intervention group (4 studies), and 20/114 (17.54%) for the WL group (4 studies). There was no significant difference between the two groups. The RR value was 0.92 (95% CI 0.52 to 1.62, *p* = 0.77, Egger’s test *P* = 0.342) with low heterogeneity (I^2^ = 3%, *p* = 0.38 Fig. 4a). Regarding acceptability between parent-only and CBT with parents, the all-cause discontinuation rates were 23/91 (25.27%) for the parent-only group(3 studies), and 12/94 (12.77%) for the CBT with parents group (3 studies). The RR value was 1.93 (95% CI 1.05 to 3.57, *p* = 0.03) with low heterogeneity (I^2^ = 0%, *p* = 0.33 Fig. 4b).

### Sensitivity analysis

**Fig. 5:**
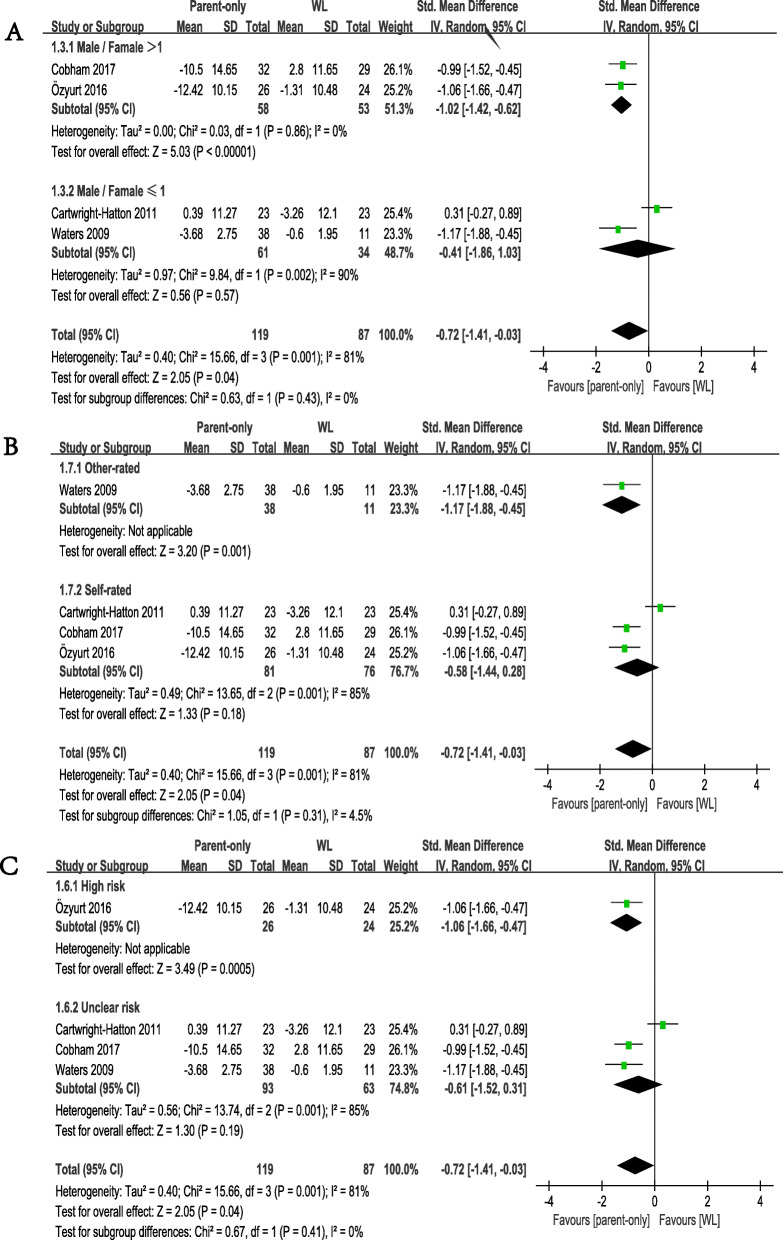
sensitivity analysis which excluded the study of Cartwright-Hatton (2011) [18]

We performed sensitivity analysis by excluding the study of Cartwright-Hatton (2011), where the effect size greatly changed (SMD=-1.05; 95%CI − 1.40 to − 0.71, ; *P*<0.00001), with low heterogeneity(I^2^ = 0%, *p* = 0.93 Fig. 5).

### Subgroup analysis

**Fig. 6:**
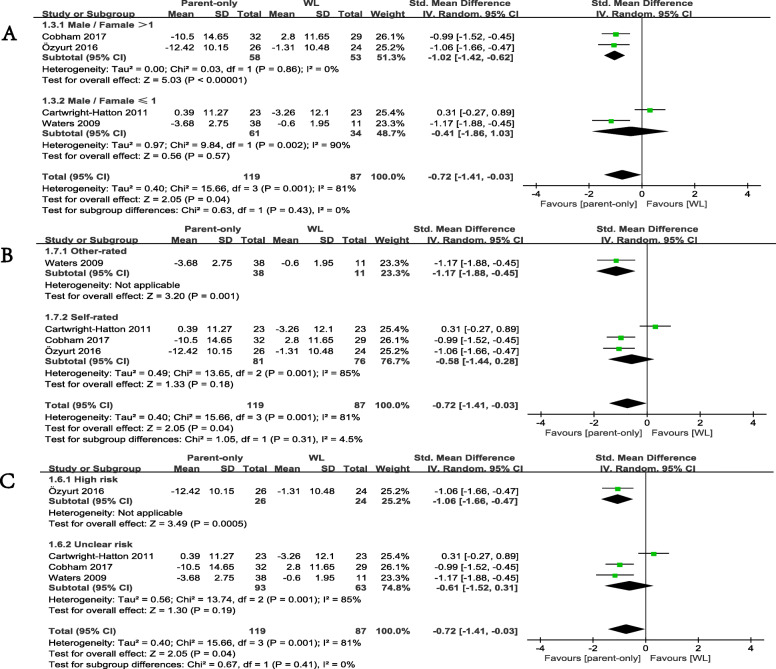
Subgroup analyses of primary efficacy outcomes, comparison of parent-only and WL. **a** gender subgroup. **b** anxiety rating scale subgroup. **c** risk of bias subgroup

We conducted subgroup analysis according to the proportion of male/female patients included in the studies. The results revealed no significant differences between two outcomes of this subgroup analysis (*p* = 0.43). Studies with more girls (male/female ≤ 1) did not show a greater effect size than WL (SMD = − 0.41, 95% CI − 1.86 to 1.03; *p* = 0.57), but studies with more boys (male/female > 1) showed a significant difference between two groups (SMD = − 1.02, 95% CI − 1.42 to − 0.62; *p* < 0.00001 Fig. 6a).

We found no significant differences between studies in which anxiety was measured using different rating scales (*p* = 0.31). When we compared parent-only CBT with WL. Studies that included patients measured with other-rated scales (SMD = − 1.17, 95% CI − 1.88 to − 0.45; *p* = 0.001) still appeared to be more effective. Studies that recruited patients measured with self-rated measures exhibited no significant differences (SMD = − 0.58, 95% CI − 1.44 to 0.28; *p* = 0.18 Fig. 6b).

Furthermore, for comparing parent-only CBT with WL, we divided previous studies into two subgroups by the risk of bias (high bias risk or unclear risk). We found no significant differences (*p* = 0.41) between studies with high risk (SMD = − 1.06, 95% CI − 1.66 to − 0.47; *p* = 0.0005) and unclear risk (SMD = − 0.61, 95% CI − 1.52 to 0.31; *p* = 0.19 Fig. 6c).

### Quality assessment

In total, the quality of included studies was relatively moderate, and the quality of studies met the standard for high risk of bias in several studies. There were three studies with a low risk of bias owing to random sequence generation. Only one study reported a low risk of bias owing to allocation concealment and performance bias. Two studies had a low risk of bias owing to detection bias. Five studies had a low risk of bias owing to reporting bias, attrition bias and other bias. The details of risk of bias are presented in Table 1 and Additional file 4.

## Discussion

Anxiety disorder is a severe disease among children and adolescents, which can threaten academic and social competence [2, 3]. Parent-only CBT is an important and novel treatment for anxiety disorder in children and adolescents. In the current study, we identified RCTs of parent-only CBT for analysis. Given the limited number of included studies, we sought to enhance the validity of our conclusions by conducting two associative pair-wise meta-analyses, including a WL control group, and comparing groups undergoing CBT with parents, and parent-only CBT. In both of the comparison groups, we assessed efficacy using mean change scores on the anxiety rating scale from baseline to post-treatment. Acceptability was represented by the proportion of participants who did not meet the standardized diagnostic criteria of anxiety disorders when treatment was finished. We then assessed remission rate and conducted subgroup analysis in the control group only.

Regarding efficacy, the results suggested that, compared with the WL control condition, parent-only CBT is an effective treatment for reducing anxiety symptoms and relieving anxiety in children, leading to remission at the end of treatment. The findings in this pair-wise meta-analysis were consistent with a previously reported network meta-analysis [30]. The network meta-analysis included studies in which more than 20% of children took psychotropic drugs, and revealed that parent-only CBT led to better outcomes than WL control condition in children with various types of anxiety disorders. Comparison revealed parent-only CBT has mild weakly (without significant difference) efficacy than CBT involving parents. According to previous studies [30, 31], CBT involving parents was effective for treating child anxiety disorders. Particularly for early childhood anxiety, CBT involving parents is reported to be more beneficial for young children than for older children because of their limited language and cognitive competence [32]. In the current study, parent-only CBT had the mild weakly (without significant difference) efficacy than CBT with parents, and was efficacious compared with the WL condition. Previous study [10] which reported child-CBT remission rate (48.47%) supported the remission of parent-only CBT (37.96%)was mild weak than child-CBT. And our results regarding the two comparison groups were consistent and in accord other child-CBT (without significant difference), suggesting that parent-only CBT is one of effective treatments for anxiety disorder in children and adolescents.

To determine the influence of various conditions on the primary efficacy outcome, we conducted subgroup analysis of male/female, high/unclear risk and self/other-rated patients, between the parent-only and WL groups. Regarding male/female differences, the results revealed more significant improvements in boys than girls. An early study of 79 children with anxiety in 1996 reported gender differences in the way parents interacted with anxious children, indicating that younger, female children benefitted more from parental involvement [33]. It is inconsistent with our finding. Therefore confirming this finding will require further research in future. Regarding high/unclear risk, excluding one high-risk study [29] changed the efficacy of the outcome. This finding highlighted the need for caution when interpreting the current results, and the importance of further studies to confirm our conclusions. When subgroups depended on self/other-rating scales, excluding one study [17] that measured anxiety in children using an other-rated scale (clinical severity rating, CSR, doctor-rated) resulted in different effects. This result may related to overstatement of improvement of anxiety symptoms by doctors. According to a previous study using three kinds of rating scales (doctor-rated, parent-rated and child-rated) to measure anxiety in the same children, the doctor-rated scale produced the least similar results among the three kinds of rating scales, while the parent-rated scale showed smaller differences than the doctor-rated scale, and the self-rated scale showed no differences [34]. This phenomenon is consistent with our speculation that findings may be overstated when doctor-rated scales are used to examine children’s anxiety.

Interestingly, for the primary outcome of efficacy(parent-only CBT compared with WL), we realized significant heterogeneity. Then we performed sensitivity analysis. We excluded the study of Cartwright-Hatton (2011), which made heterogeneity fromdwon to and *p*-value from to. We investigated deeply into the study of Cartwright-Hatton (2011). we found that the children relatively young and MASC was adopted. But MASC was developed for children 8 years and older. The self-rated scale MASC may do not inappropriate to anxiety in young children. For example, the studies (Waters 2009 and Monga 2015) included young children adopted other-rated scale.

Regarding acceptability, we found no significant differences between the parent-only CBT and WL conditions. However, more families tended to drop out of treatment in the parent-only group compared with the CBT with parents group. This result is similar to those of other studies investigating internet-based delivery and bibliotherapy as alternative modes of CBT treatment [35–37]. It is possible that parents did not have sufficient trust in the efficacy of parent-only CBT because their children were not directly involved in the treatment, resulting in a tendency to drop out early.. And the results of a previous study suggest that the additional responsibility in the parent-only condition may explain the tendency to drop out in the parent-only condition. This explanation supports the importance of assessing the level of sense of responsibility of parents and enhancing it prior to commencing treatment [17]. However, the conclusions that can be drawn from the current findings regarding acceptability are limited due to the small sample size.

In the current study, we did not investigate the effects of parent-only CBT in young children and older children, respectively, due to a lack of studies. According to previous studies of CBT with parents in young children [32], family-based CBT in which parents are highly involved is a well-established effective intervention for early childhood anxiety. Thus, future studies should investigate whether parent-only CBT is a similarly effective treatment, which could be more beneficial for young children than the older. Moreover, in the etiology of childhood anxiety, the influence of parents’ own anxiety levels is important and well recognized [38]. In the current meta-analysis, only two studies reported scale score change of parents’ own anxiety levels from pre-treatment to post-treatment. Waters reported that parents with high anxiety scores were more likely to drop out, both in the intervention group and the control group, but only a non-significant trend was observed on the DASS-42 Anxiety subscales from pre-treatment to post-treatment [17]. And Ozyurt reported a significant improvement in parental anxiety in the intervention group from pre-treatment to post-treatment [29]. No previous studies have explored the differences between implementing parent-only CBT for children with anxiety when parents met the diagnostic criteria for anxiety or depression. Elucidating these issues will require more RCTs in future.

The current study involved several limitations that should be considered. First, the sample size was not sufficient to ensure reliable statistical power, particularly in subgroup analyses. In addition, the unsatisfactory quality of some of the included studies limited the reliability of the conclusions, and the studies included in the review exhibited substantial heterogeneity. Therefore, caution is required when interpreting the current findings. Moreover, other types of CBT involving parental participation were not taken into consideration, such as parent-delivered CBT, internet-delivered CBT with parents, and telephone-delivered CBT with parents. We did not investigate the efficacy of parent-only CBT for specific types of anxiety in children. Elucidating these issues will require further investigation. Due to a lack of data in the included studies, we did not examine follow-up assessment results. Resolving this shortcoming will require future studies.

## Conclusions

Our meta-analysis suggests that parent-only CBT is one of effective interventions for children with anxiety disorder. Although parent-only CBT increased the rate of all-cause discontinuation compared with CBT with parents, it did not lead to more patients dropping out compared with the WL control group. Current evidence indicates that parent-only CBT is both an acceptable and alternative treatment compared with a control condition for children and adolescents with anxiety disorder.

## Supplementary Information

**Additional file 1: Table S1-S6.** Literature search report: Cochrane (613), Embase (386), ProQuest (810), PsycINFO (559), PubMed (1393), Web of Science (3118) and Additional file 2

**Additional file 2.** 112 excluded studies, and the reasons they were excluded.

**Additional file 3.** Data extraction forms.

**Additional file 4.** Graphical overview of the methodological quality of included studies.

## Data Availability

All data generated or analyzed during this study are included in this article.

## Abbreviations

AC: Allocation concealment
ADIS: Anxiety Disorders Interview Schedule for DSM-4
BOA: Blinding of outcome assessment
BPP: Blinding of participants and personnel
CBCL: Child Behavior Checklist
CBT: Cognitive behavioral therapy
CIs: Confidence intervals
CSR: Clinician severity ratings
DASS: Depression Anxiety Stress Scale
DSM: Diagnostic and Statistical Manual of Mental Disorders
FSSC-R: Fear Survey for Children Revised
IOD: Incomplete outcome data
MASC: Multidimensional Anxiety Scale for Children
OB: Other bias
RCMAS: Revised Children’s Manifest Anxiety Scale
RCT: Randomized controlled trial
RRs: Risk ratios
RSG: Random sequence generation
SADS: Schedule for Affective Disorders and Schizophrenia
SCARED: Screen for Anxiety and Related Disorders
SCAS: Spence Children’s Anxiety Scale
SMD: Standardized mean difference
SPAI-C: Social Phobia and Anxiety Inventory for Children
SR: Selective reporting
WL: Waitlist
