# Correction to: Efficacy and acceptability of parent-only group cognitive behavioral intervention for treatment of anxiety disorder in children and adolescents: a meta-analysis of randomized controlled trials

**DOI:** 10.1186/s12888-021-03075-8

**Published:** 2021-02-16

**Authors:** Bangmin Yin, Teng Teng, Lyu Tong, Xuemei Li, Li Fan, Xinyu Zhou, Peng Xie

**Affiliations:** 1 Department of Neurology, People’s Hospital of Deyang City, Deyang, China; 2 Department of Neurology, The First Affiliated Hospital of Chongqing Medical University, Chongqing, China; 3 NHC Key Laboratory of Diagnosis and Treatment on Brain Functional Diseases, The First Affiliated Hospital, Chongqing Medical University, No. 1 Youyi Road, Chongqing, 400016 China; 4 Department of Immunology, College of Basic Medical Sciences, China Medical University, Shenyang, China; 5 Department of Psychiatry, The First Affiliated Hospital of Chongqing Medical University, No. 1 Youyi Road, Chongqing, 400016 China

**Correction to: BMC Psychiatry 21, 29 (2021)**

**https://doi.org/10.1186/s12888-020-03021-0**

Following publication of the original article [1], the authors identified errors in Fig. 5 and Fig. 6. The correct figures are given below.

**Fig. 5:**
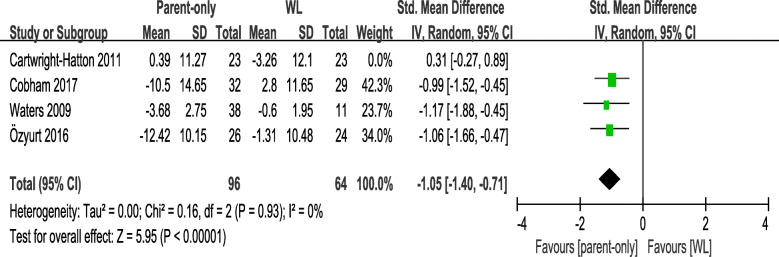
sensitivity analysis which excluded the study of Cartwright-Hatton (2011) [18]

**Fig. 6:**
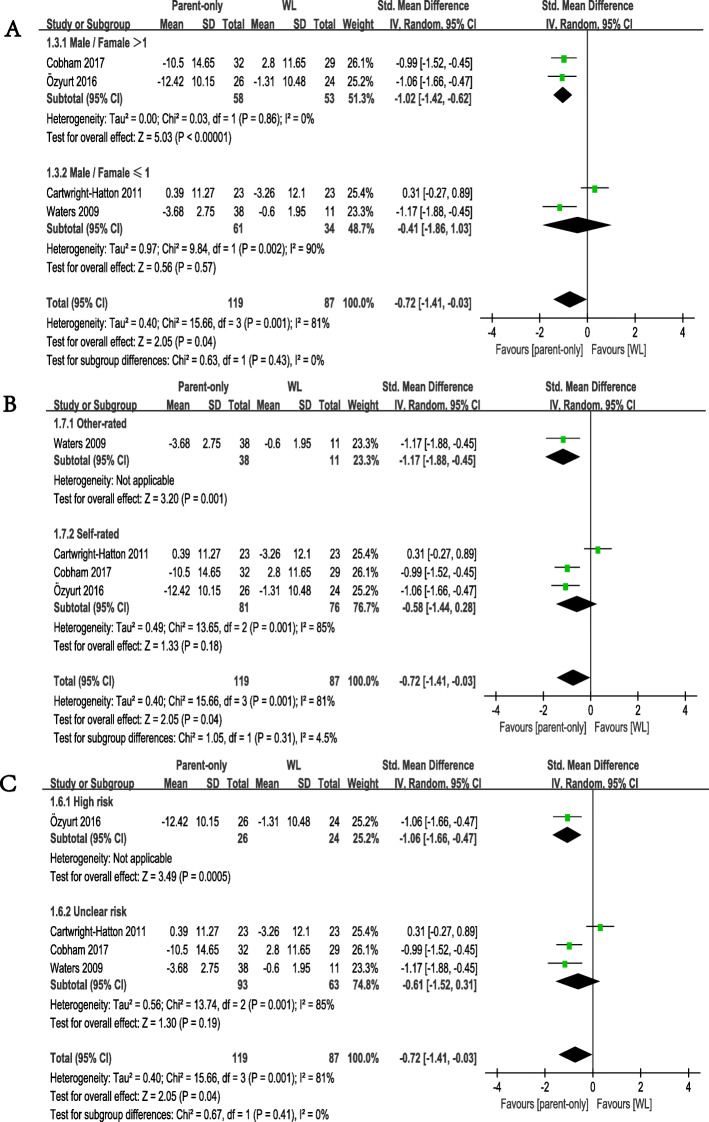
Subgroup analyses of primary efficacy outcomes, comparison of parent-only and WL. **a** gender subgroup. **b** anxiety rating scale subgroup. **c** risk of bias subgroup

The author group has been updated above and the original article [1] has been corrected.
