# Supplementary material for: Efficacy and acceptability of parent-only group cognitive behavioral intervention for treatment of anxiety disorder in children and adolescents: a meta-analysis of randomized controlled trials
Source: BMC Psychiatry. 2021 Jan 11;21:29. doi: 10.1186/s12888-020-03021-0 (PMC7802251; doi:10.1186/s12888-020-03021-0)
Supplement: Supplementary file 1 — Additional file 1: Table S1-S6. Literature search report: Cochrane (613), Embase (386), ProQuest (810), PsycINFO (559), PubMed (1393), Web of Science (3118) and Additional file 2 [file 12888_2020_3021_MOESM1_ESM.docx]

Efficacy and Acceptability of Parent-only Group Cognitive Behavioral Intervention for Treatment of Anxiety disorder in Children and Adolescents : A Meta-analysis of Randomized Controlled Trials

Bangmin Yin^12^, Teng Teng^12^, Lyu Tong^3^, Xuemei Li^12^, Li Fan^12^, Xinyu Zhou^3*^, Peng Xie^12*^

The article contains supplementary Table S1-S6.

Table S1

| Literature Search Report | | | | | | |
| --- | --- | --- | --- | --- | --- | --- |
| Database | [cochrance](http://apps.webofknowledge.com/home.do?SID=D5CEfU6wEFtURGDD6co) | Search Time | 20190623 | Search Name | | BY |
| Search process | | | | | | |
| Search formula | | | limited | | Search result | |
| #1 anxiety OR anxious OR phobic OR fear OR fears OR phobia OR phobias OR “panic disorder” OR “overanxious disorder” OR “avoidant disorder” OR agoraphobia OR “selective mutism” OR “panic attack speciﬁer” OR “combat disorder” OR “mixed disorder” OR neurosis OR neuroses OR neurotic OR “school refusal” in Title Abstract Keyword | | | Trials | | 47336 | |
| #2 adolesc OR child OR boy OR girl OR juvenil OR minors OR paediatri OR pediatri OR pubescen OR school OR student OR teen OR young OR youth OR class OR preschool OR pre-school in Title Abstract Keyword | | | Trials | | 258959 | |
| #3  behavio* OR cogniti OR CBT behavio* OR cogniti OR CBT in Title Abstract Keyword | | | Trials | | 88660 | |
| #4  parent OR mother OR father in Title Abstract Keyword | | | Trials | | 21884 | |
| #5 random OR allocate OR “cross over” OR controlled in Title Abstract Keyword | | | Trials | | 679129 | |
| #6 #1 AND #2 AND #3 AND #4 AND #5 | | | Trials | | 613 | |
| Total | | | | | 613 | |

Table S2

| Literature Search Report | | | | | | |
| --- | --- | --- | --- | --- | --- | --- |
| Database | [embase](http://apps.webofknowledge.com/home.do?SID=D5CEfU6wEFtURGDD6co) | Search Time | 20190623 | Search Name | | BY |
| Search process | | | | | | |
| Search formula | | | limited | | Search result | |
| #1 anxiety:ab,ti OR anxious:ab,ti OR phobic:ab,ti OR fear:ab,ti OR fears:ab,ti OR phobia:ab,ti OR phobias:ab,ti OR 'panic disorder':ab,ti OR 'overanxious disorder':ab,ti OR 'avoidant disorder':ab,ti OR agoraphobia:ab,ti OR 'selective mutism':ab,ti OR 'panic attack speciﬁer':ab,ti OR 'combat disorder':ab,ti OR 'mixed disorder':ab,ti OR neurosis:ab,ti OR neuroses:ab,ti OR neurotic:ab,ti OR 'school refusal':ab,ti | | |  | | [351,413](https://www.embase.com/) | |
| #2 adolesc:ab,ti OR child:ab,ti OR boy:ab,ti OR girl:ab,ti OR juvenil:ab,ti OR minors:ab,ti OR paediatri:ab,ti OR pediatri:ab,ti OR pubescen:ab,ti OR school:ab,ti OR student:ab,ti OR teen:ab,ti OR young:ab,ti OR youth:ab,ti OR class:ab,ti OR preschool:ab,ti OR 'pre school':ab,ti | | |  | | [1,948,747](https://www.embase.com/) | |
| #3 behavio*:ab,ti OR cogniti:ab,ti OR cbt:ab,ti  behavio*:ab,ti OR cogniti:ab,ti OR cbt:ab,ti | | |  | | [1,336,152](https://www.embase.com/) | |
| #4 parent:ab,ti OR mother:ab,ti OR father:ab,ti | | |  | | [311,802](https://www.embase.com/) | |
| #5 random:ab,ti OR allocate:ab,ti OR 'cross over':ab,ti OR controlled:ab,ti | | |  | | [1,210,226](https://www.embase.com/) | |
| #6 #2 AND #3 AND #4 AND #5 AND #6 | | |  | | [386](https://www.embase.com/) | |
| Total | | | | | 386 | |

Table S3

| Literature Search Report | | | | | | |
| --- | --- | --- | --- | --- | --- | --- |
| Database | ProQuest | Search Time | 20190623 | Search Name | | BY |
| Search process | | | | | | |
| Search formula | | | limited | | Search result | |
| #1 ab(anxiety OR anxious OR phobic OR fear OR fears OR phobia OR phobias OR “panic disorder” OR “overanxious disorder” OR “avoidant disorder” OR agoraphobia OR “selective mutism” OR “panic attack speciﬁer” OR “combat disorder” OR “mixed disorder” OR neurosis OR neuroses OR neurotic OR “school refusal”) | | |  | | 486,945 | |
| #2 ab(adolesc OR child OR boy OR girl OR juvenil OR minors OR paediatri OR pediatri OR pubescen OR school OR student OR teen OR young OR youth OR class OR preschool OR pre-school) | | |  | | 6,643,461 | |
| #3 ab(behavio* OR cogniti OR CBT ) | | |  | | 4,136,063 | |
| #4 ab(parent OR mother OR father) | | |  | | 1,061,811 | |
| #5 ab(random OR allocate OR “cross over” OR controlled) | | |  | | 2,837,212 | |
| #6 #1 AND #2 AND #3 AND #4 AND #5 | | |  | | 810 | |
| Total | | | | | 810 | |

Table S4

| Literature Search Report | | | | | | |
| --- | --- | --- | --- | --- | --- | --- |
| Database | PsycINFO | Search Time | 20190623 | Search Name | | BY |
| Search process | | | | | | |
| Search formula | | | limited | | Search result | |
| 1 (anxiety or anxious or phobic or fear or fears or phobia or phobias or panic disorder or overanxious disorder or avoidant disorder or agoraphobia or selective mutism or panic attack or combat disorder or mixed disorder or neurosis or neuroses or neurotic or school refusal).mp. [mp=title, abstract, heading word, table of contents, key concepts, original title, tests & measures, mesh] | | |  | | 314943 | |
| 2 limit 1 to abstracts | | |  | | 307016 | |
| 3 (adolesc or child or boy or girl or juvenil or minors or paediatri or pediatri or pubescen or school or student or teen or young or youth or class or preschool or pre-school).mp. [mp=title, abstract, heading word, table of contents, key concepts, original title, tests & measures, mesh] | | |  | | 1106354 | |
| 4 limit 3 to abstracts | | |  | | 1048739 | |
| 5 (behavio* or cogniti or CBT).mp. [mp=title, abstract, heading word, table of contents, key concepts, original title, tests & measures, mesh] | | |  | | 1190907 | |
| 6  limit 5 to abstracts | | |  | | 1151764 | |
| 7 (parent or mother or father).mp. [mp=title, abstract, heading word, table of contents, key concepts, original title, tests & measures, mesh] | | |  | | 187876 | |
| 8 limit 7 to abstracts | | |  | | 179945 | |
| 9 (random or allocate or cross over or controlled).mp. [mp=title, abstract, heading word, table of contents, key concepts, original title, tests & measures, mesh] | | |  | | 176725 | |
| 10 limit 9 to abstracts | | |  | | 176104 | |
| 11 2 and 4 and 6 and 8 and 10 | | |  | | 559 | |
| Total | | | | | 559 | |

Table S5

| Literature Search Report | | | | | | |
| --- | --- | --- | --- | --- | --- | --- |
| Database | Pubmed | Search Time | 20190623 | Search Name | | BY |
| Search process | | | | | | |
| Search formula | | | limited | | Search result | |
| #1 Search (anxiety[Title/Abstract] OR anxious[Title/Abstract] OR phobic[Title/Abstract] OR fear[Title/Abstract] OR fears[Title/Abstract] OR phobia[Title/Abstract] OR phobias[Title/Abstract] OR “panic disorder*”[Title/Abstract] OR “overanxious disorder*”[Title/Abstract] OR “avoidant disorder*”[Title/Abstract] OR agoraphobia[Title/Abstract] OR “selective mutism”[Title/Abstract] OR “panic attack speciﬁer”[Title/Abstract] OR “combat disorder*”[Title/Abstract] OR “mixed disorder*”[Title/Abstract] OR neurosis[Title/Abstract] OR neuroses[Title/Abstract] OR neurotic[Title/Abstract] OR “school refusal”[Title/Abstract]) | | |  | | [254852](https://www.ncbi.nlm.nih.gov/pubmed/?cmd=HistorySearch&querykey=1) | |
| #2 Search anxiety disorders[MeSH Major Topic] | | |  | | [55925](https://www.ncbi.nlm.nih.gov/pubmed/?cmd=HistorySearch&querykey=3) | |
| #3 #1 or #2 | | |  | | [2700340](https://www.ncbi.nlm.nih.gov/pubmed/?cmd=HistorySearch&querykey=15) | |
| #4 Search (adolesc*[Title/Abstract] OR child*[Title/Abstract] OR boy*[Title/Abstract] OR girl*[Title/Abstract] OR juvenil*[Title/Abstract] OR minors[Title/Abstract] OR paediatri*[Title/Abstract] OR pediatri*[Title/Abstract] OR pubescen*[Title/Abstract] OR school*[Title/Abstract] OR student*[Title/Abstract] OR teen*[Title/Abstract] OR young[Title/Abstract] OR youth*[Title/Abstract] OR class*[Title/Abstract] OR preschool[Title/Abstract] OR pre-school[Title/Abstract]) | | |  | | [3547839](https://www.ncbi.nlm.nih.gov/pubmed/?cmd=HistorySearch&querykey=4) | |
| #5 Search  (behavio*[Title/Abstract] OR cogniti*[Title/Abstract] OR CBT[Title/Abstract] OR famil*[Title/Abstract] OR “contingency management”[Title/Abstract]) | | |  | | 2374401 | |
| #6 Search (parent*[Title/Abstract] OR mother[Title/Abstract] OR father[Title/Abstract]) | | |  | | [491707](https://www.ncbi.nlm.nih.gov/pubmed/?cmd=HistorySearch&querykey=20) | |
| #7 #5AND#6 | | |  | | [152516](https://www.ncbi.nlm.nih.gov/pubmed/?cmd=HistorySearch&querykey=21) | |
| #8 #3 AND #4 AND #7 | | |  | | [9567](https://www.ncbi.nlm.nih.gov/pubmed/?cmd=HistorySearch&querykey=22) | |
| #9 Search (random*[Title/Abstract] OR allocate*[Title/Abstract] OR assign*[Title/Abstract] OR “cross over*”[Title/Abstract] OR crossover*[Title/Abstract] OR controlled[Title/Abstract]) | | |  | | [1703138](https://www.ncbi.nlm.nih.gov/pubmed/?cmd=HistorySearch&querykey=47) | |
| #8 AND #9 | | |  | | [1393](https://www.ncbi.nlm.nih.gov/pubmed/?cmd=HistorySearch&querykey=48) | |
| Total | | | | | 1393 | |

Table S6

| Literature Search Report | | | | | | |
| --- | --- | --- | --- | --- | --- | --- |
| Database | [Web of Science](http://apps.webofknowledge.com/home.do?SID=D5CEfU6wEFtURGDD6co) | Search Time | 20190623 | Search Name | | BY |
| Search process | | | | | | |
| Search formula | | | limited | | Search result | |
| #1 TS=(adolesc OR child OR boy OR girl OR juvenil OR minors OR paediatri OR pediatri OR pubescen OR school OR student OR teen OR young OR youth OR class OR preschool OR pre-school)  *索引=SCI-EXPANDED, SSCI, A&HCI, CPCI-S, CPCI-SSH, ESCI 时间跨度=所有年份* | | |  | | [4,297,765](http://apps.webofknowledge.com/summary.do?product=WOS&doc=1&qid=71&SID=D5CEfU6wEFtURGDD6co&search_mode=AdvancedSearch&update_back2search_link_param=yes) | |
| #2 TS=(anxiety OR anxious OR phobic OR fear OR fears OR phobia OR phobias OR “panic disorder” OR “overanxious disorder” OR “avoidant disorder” OR agoraphobia OR “selective mutism” OR “panic attack speciﬁer” OR “combat disorder” OR “mixed disorder” OR neurosis OR neuroses OR neurotic OR “school refusal”)  *索引=SCI-EXPANDED, SSCI, A&HCI, CPCI-S, CPCI-SSH, ESCI 时间跨度=所有年份* | | |  | | [343,407](http://apps.webofknowledge.com/summary.do?product=WOS&doc=1&qid=70&SID=D5CEfU6wEFtURGDD6co&search_mode=AdvancedSearch&update_back2search_link_param=yes) | |
| #3 TS=(behavio* OR cogniti OR CBT)  *索引=SCI-EXPANDED, SSCI, A&HCI, CPCI-S, CPCI-SSH, ESCI 时间跨度=所有年份* | | |  | | [3,448,785](http://apps.webofknowledge.com/summary.do?product=WOS&doc=1&qid=82&SID=D5CEfU6wEFtURGDD6co&search_mode=AdvancedSearch&update_back2search_link_param=yes) | |
| #4 TS=(parent OR mother OR father)  *索引=SCI-EXPANDED, SSCI, A&HCI, CPCI-S, CPCI-SSH, ESCI 时间跨度=所有年份* | | |  | | [569,133](http://apps.webofknowledge.com/summary.do?product=WOS&doc=1&qid=84&SID=D5CEfU6wEFtURGDD6co&search_mode=AdvancedSearch&update_back2search_link_param=yes) | |
| #5 TS=(random OR allocate OR “cross over” OR controlled)  *索引=SCI-EXPANDED, SSCI, A&HCI, CPCI-S, CPCI-SSH, ESCI 时间跨度=所有年份* | | |  | | [6,285,796](http://apps.webofknowledge.com/summary.do?product=WOS&doc=1&qid=85&SID=D5CEfU6wEFtURGDD6co&search_mode=AdvancedSearch&update_back2search_link_param=yes) | |
| #6 #1 AND #2 AND #3 AND #4 AND #5  *索引=SCI-EXPANDED, SSCI, A&HCI, CPCI-S, CPCI-SSH, ESCI 时间跨度=所有年份* | | |  | | [3,118](http://apps.webofknowledge.com/summary.do?product=WOS&doc=1&qid=86&SID=D5CEfU6wEFtURGDD6co&search_mode=CombineSearches&update_back2search_link_param=yes) | |
| Total | | | | | 3118 | |
