# Supplementary material for: Efficacy and acceptability of parent-only group cognitive behavioral intervention for treatment of anxiety disorder in children and adolescents: a meta-analysis of randomized controlled trials
Source: BMC Psychiatry. 2021 Jan 11;21:29. doi: 10.1186/s12888-020-03021-0 (PMC7802251; doi:10.1186/s12888-020-03021-0)
Supplement: Supplementary file 4 — Additional file 4. Graphical overview of the methodological quality of included studies. [file 12888_2020_3021_MOESM4_ESM.pdf]

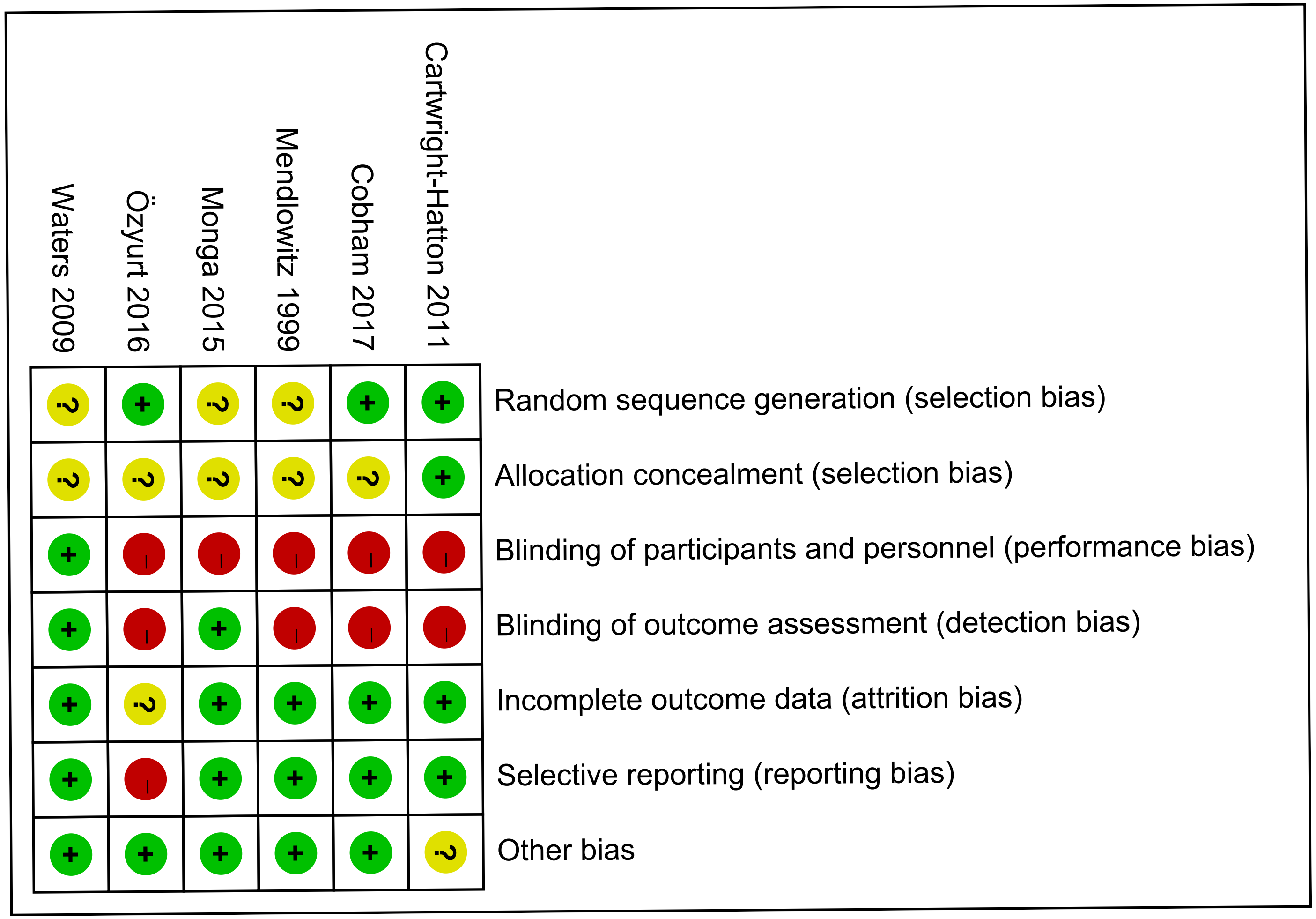

Waters 2009
Özyurt 2016
Monga 2015
Mendlowitz 1999
Cobham 2017
Cartwright-Hatton 2011
Random sequence generation (selection bias)
Allocation concealment (selection bias)
Blinding of participants and personnel (performance bias)
Blinding of outcome assessment (detection bias)
Incomplete outcome data (attrition bias)
Selective reporting (reporting bias)
Other bias
